# Lipid-Adjusted Vitamin E Status in Children with Chronic Liver Disease: Reclassification Patterns and Differences According to Cholestasis and Liver Transplantation

**DOI:** 10.3390/nu18193283

**Published:** 2026-10-06

**Authors:** Sorina Adam, Alina Grama, Alexandra Mititelu, Gabriel Bența, Răzvan M. Cherecheș, Tudor Lucian Pop

**Affiliations:** 1 “Iuliu Haţieganu” University of Medicine and Pharmacy, 400012 Cluj-Napoca, Romania; adam.rogozan.sorina@elearn.umfcluj.ro; 2 2nd Pediatric Discipline, Department of Mother and Child, Faculty of Medicine, “Iuliu Haţieganu” University of Medicine and Pharmacy, 400012 Cluj-Napoca, Romania; perta.alexandra@elearn.umfcluj.ro (A.M.); benta.gabriel@elearn.umfcluj.ro (G.B.); tudor.pop@umfcluj.ro (T.L.P.); 3 2nd Pediatric Clinic, Emergency Clinical Hospital for Children, 400124 Cluj-Napoca, Romania; 4 HIVE Health Innovation Center, Babeș-Bolyai University, 400095 Cluj-Napoca, Romania; razvan.chereches@ubbcluj.ro

**Keywords:** vitamin E, α-tocopherol, pediatric chronic liver disease, cholestasis, lipid-adjusted vitamin E, vitamin E-to-cholesterol ratio, fat-soluble vitamins, liver transplantation

## Abstract

Background: Serum α-tocopherol depends on lipids, so absolute concentrations misclassify vitamin E status in children with chronic liver disease (CLD). The CALIPER program defined lipid-adjusted E:cholesterol (E:C) and E:triglyceride (E:TG) ratios, but their application in mixed-etiology pediatric CLD using immunoassay has not been reported. Methods: Exploratory single-center cross-sectional study of 66 children (observed age range 4 months–17.9 years) with mixed-etiology CLD. Retinol and total vitamin E were measured by ELISA, whereas the CALIPER reference intervals applied here were established for α-tocopherol measured by HPLC; the two platforms are not interchangeable, so every classification reported is an exploratory application of those cutoffs. Vitamin E status was classified by absolute concentration, E:C (<3.7) and E:TG (<8.5). Cholestatic (*n* = 15), non-cholestatic (*n* = 45) and post-transplant (*n* = 6) groups were compared. Results: Vitamin A was largely preserved (5.2% below WHO). Vitamin E was low in 45.3% by absolute concentration, 62.5% by E:C and 43.8% by E:TG. Of 35 children with a normal absolute vitamin E concentration, 11 (31.4%) were reclassified as low by E:C. Low E:C differed across groups (cholestatic 78.6%, non-cholestatic 63.6%, post-transplant 16.7%; *p* = 0.030). E:TG was inversely correlated with 25(OH)D (rho = −0.45; *p* < 0.001). Conclusions: Lipid-adjusted interpretation reclassified a substantial proportion of children whose absolute vitamin E concentration appeared adequate, and the prevalence of a low E:C ratio was lower among post-transplant participants than in the other two groups. In this exploratory cohort, the E:C ratio showed greater potential than the E:TG ratio for lipid-adjusted interpretation of vitamin E status; prospective validation against HPLC-based measurements and established functional indices is required before routine clinical adoption.

## 1. Introduction

Vitamins A and E are fat-soluble micronutrients essential for vision, immune defense, neurological development, and antioxidant protection in children [1,2]. The liver is central to their handling: it is the main site of storage and metabolism and synthesizes the transport proteins that determine their bioavailability [1,3,4]. In chronic liver disease (CLD), reduced bile acid secretion and impaired enterohepatic circulation cause fat malabsorption and progressive depletion of fat-soluble vitamin stores. Published pediatric series reported vitamin A deficiency in 67–77% and vitamin E deficiency in 41–91% of children with cholestatic CLD, compared with approximately 37% and 10.5%, respectively, in non-cholestatic CLD [2,5,6].

Among the fat-soluble vitamins, vitamin E (α-tocopherol) is the most vulnerable to depletion in cholestatic conditions, because of its high hydrophobicity and its complete dependence on micellar solubilization for intestinal absorption [1,7]. When intraluminal bile acid concentrations fall below the critical micellar concentration, vitamin E absorption is severely impaired, and tissue stores decline [3,7]. If untreated, deficiency causes a progressive neuromuscular syndrome with areflexia, cerebellar ataxia, ophthalmoplegia, and, in infants, hemolytic anemia [8,9]. Because the neurological consequences are potentially irreversible, early detection is essential.

The interpretation of serum vitamin E is complicated by its lipoprotein dependence. Because α-tocopherol circulates almost entirely within lipoproteins, the hyperlipidemia characteristic of cholestasis can produce apparently normal serum concentrations even when tissue stores are depleted. Sokol and colleagues first demonstrated this discrepancy in children with chronic cholestasis: 3 of 11 patients (27%) with histopathologically confirmed vitamin E deficiency on sural nerve biopsy nonetheless had absolute serum α-tocopherol within the reference range [7]. These findings led to the development of lipid-adjusted indices, initially the ratio of α-tocopherol to total lipids proposed by Horwitt and Sokol [7,10], and subsequently the simpler and more accessible ratios to cholesterol (E:C) and to triglycerides (E:TG), which have been evaluated in adults and children with a range of conditions [11].

The CALIPER (Canadian Laboratory Initiative on Pediatric Reference Intervals) program established age-specific reference intervals for vitamins A and E in a cohort of 342 healthy children from birth to 19 years, and additionally defined normative ranges for the E:C and E:TG ratios [12]. These ratios can be calculated from a standard lipid panel and do not require the phospholipid quantification needed for the original Sokol formula. Their application in pediatric CLD, however, has been reported mainly in single-etiology cohorts, most often biliary atresia, which limits the generalisability of prior findings [5].

Vitamin A status in CLD is governed by partly distinct mechanisms. Dietary retinol requires bile-dependent micellar absorption; thus, cholestasis reduces uptake via the same route as for vitamin E [2,3]. In addition, hepatocellular dysfunction impairs the synthesis of retinol-binding protein (RBP4), which is required to mobilize retinol from hepatic stores, and RBP4 is a negative acute-phase reactant whose circulating levels are further suppressed during chronic inflammation [13]. Serum retinol is homeostatically regulated and remains within range until hepatic stores are markedly depleted, so the WHO threshold of 0.70 µmol/L identifies advanced rather than early deficiency [14].

A practical limitation of many pediatric centers is that HPLC analysis of retinol and α-tocopherol is not routinely available. Enzyme-linked immunosorbent assays (ELISA) are widely available but have not been systematically evaluated in pediatric CLD. Because the CALIPER reference values were established by HPLC, applying them to ELISA-derived measurements is an exploratory step; its analytical implications are set out in the Methods and the Discussion.

Serum vitamin D has been inversely correlated with liver stiffness measured by shear wave elastography in children after Kasai portoenterostomy [15], but no equivalent analysis has been reported for vitamins A or E using lipid-adjusted indices in a mixed-etiology pediatric CLD cohort. The relationship between fat-soluble vitamin status and the severity of fibrosis has also not been systematically investigated using these indices.

The primary question of this exploratory study concerns vitamin E; vitamin A was examined as a complementary analysis. The aims were (i) to describe vitamin E status, and complementarily vitamin A status, in a mixed-etiology pediatric CLD cohort using immunoassay-based measurement and age-stratified CALIPER reference intervals; (ii) to quantify the extent to which lipid-adjusted interpretation of vitamin E reclassifies children compared with the absolute vitamin E concentration; (iii) to perform a pre-specified secondary analysis across three clinically meaningful groups (cholestatic CLD, non-cholestatic CLD and post-transplant); and (iv) to examine relationships between vitamin E indices and biochemical markers of disease severity, hepatic fibrosis and serum vitamin D. Because measurement was performed by immunoassay rather than HPLC, the study is framed as exploratory: the primary interpretive question is whether lipid-adjusted indices change how children are classified, not whether the absolute prevalence of deficiency is precisely quantified.

## 2. Materials and Methods

### 2.1. Study Design and Population

**Figure 1:**
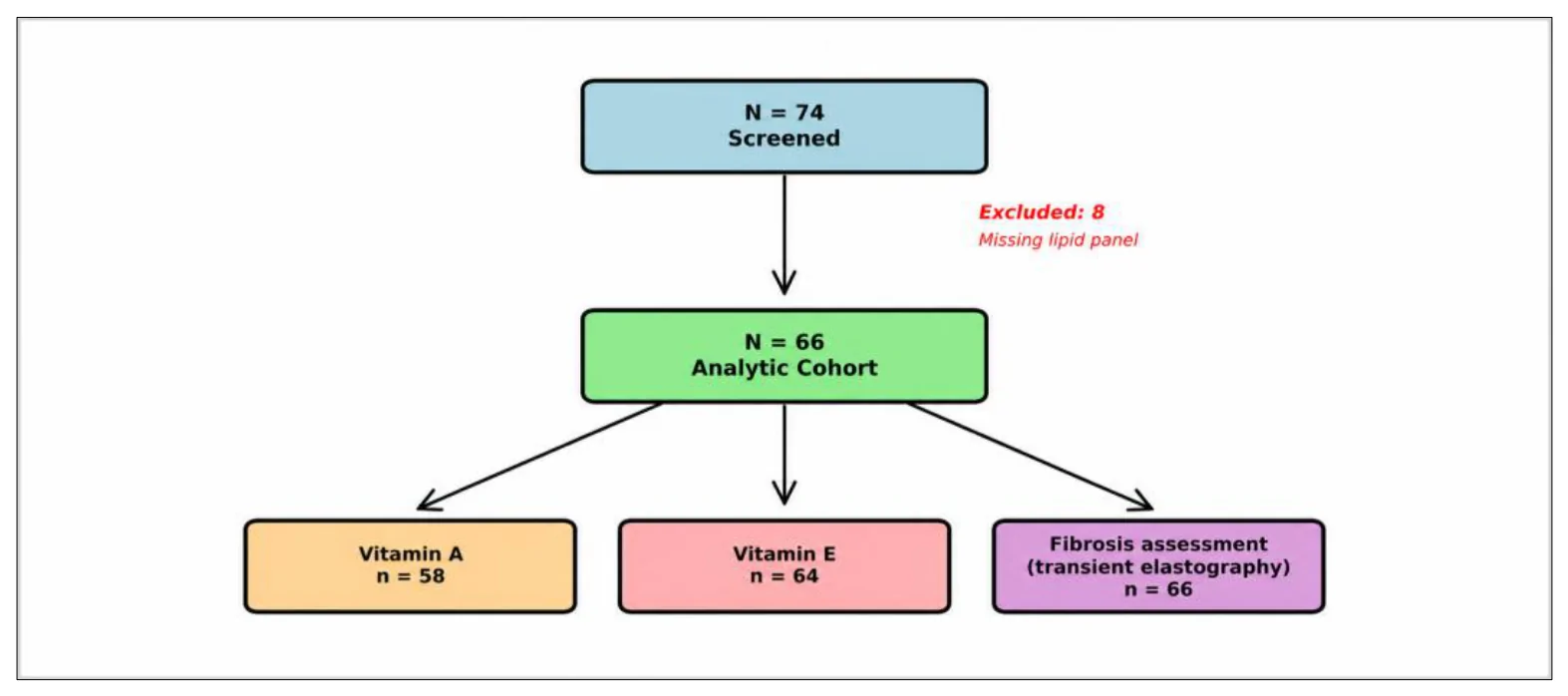
Patient Flow Diagram. Of 74 children screened for chronic liver disease between January 2023 and November 2024, eight were excluded due to missing lipid panel data, resulting in an analytic cohort of 66. Serum vitamin A was measurable in 58 children, vitamin E in 64, and transient elastography (FibroScan) was available for all 66.

This was an exploratory, single-center, observational cross-sectional study conducted at the 2nd Pediatric Clinic, Emergency Clinical Hospital for Children, Cluj-Napoca, Romania, between January 2023 and November 2024. Children aged 2 months to 18 years with established CLD of any etiology followed at the center during the study period were eligible; the observed age range of the 66 children enrolled was 4 months to 17.9 years. Because the pre-specified indices required a fasting lipid panel, children without contemporaneous total cholesterol and triglycerides were excluded. Transient elastography was required to assess fibrosis. Of 74 children initially screened, 8 were excluded because of an incomplete lipid panel, leaving an analytic cohort of 66 (Figure 1).

The study was approved by the Ethics Committee of the Emergency Clinical Hospital for Children, Cluj-Napoca (approval number 94/28.07.2023) and was conducted in accordance with the Declaration of Helsinki. Written informed consent was obtained from parents or legal guardians and assent from children aged seven years or older.

### 2.2. Clinical and Biochemical Assessment

Patients were assessed at a single study visit. Fasting venous blood was drawn after an overnight fast of at least 8 to 12 h into serum separator tubes. Total and direct bilirubin, gamma-glutamyltransferase (GGT), cholinesterase, total cholesterol, and triglycerides were measured by standardized automated methods on a Mindray BS-2800M biochemistry analyzer (Mindray Bio-Medical Electronics, Shenzhen, China). Serum albumin, prothrombin time, and 25-hydroxyvitamin D were measured on a Mindray CL-8000i chemiluminescence immunoassay analyzer at the same visit. All laboratory determinations were performed at the certified laboratory of the Emergency Clinical Hospital for Children, Cluj-Napoca. Liver fibrosis was staged by transient elastography using a FibroScan device (Echosens, Paris, France) with pediatric-appropriate probe selection and quality criteria (at least 10 valid measurements, interquartile range to median ratio below 30%), by an experienced operator, and expressed as METAVIR stage F0 to F4 using etiology-adjusted cutoffs.

Etiological categories were autoimmune hepatitis, cholestatic disease (biliary atresia post-Kasai, Alagille syndrome [16], progressive familial intrahepatic cholestasis, other chronic cholestatic disorders) [17], chronic hepatitis B, Wilson disease, metabolic disease, vascular disease, and post-liver transplantation. For the pre-specified secondary analysis, children were grouped into three clinically meaningful categories: (i) biochemical cholestasis, defined per the ESPGHAN/NASPGHAN 2017 criteria as conjugated (direct) bilirubin above 1.0 mg/dL when total bilirubin was below 5.0 mg/dL, or above 20% of total when total was 5.0 mg/dL or higher [18]; (ii) non-cholestatic CLD; and (iii) post-transplant recipients. In this cohort, the biochemical and the etiological (clinical) definitions of cholestasis identified the same 15 children. This complete overlap is expected given that biochemical cholestasis is, by definition, a feature of the underlying cholestatic disorders included in the etiological category, and no children with non-cholestatic etiologies met the ESPGHAN biochemical criterion at the study visit. The two definitions are not independent constructs in the analysis and are reported together for transparency.

### 2.3. Vitamin A and Vitamin E Measurement

Serum retinol (vitamin A) was measured using a competitive ELISA (Vitamin A ELISA Kit, Cat. No. E-EL-0135, Elabscience Bionovation Inc., Wuhan, China) according to the manufacturer’s instructions. The assay is based on competitive inhibition with a tetramethylbenzidine (TMB) substrate, and absorbance was read at 450 nm. The analytical measurement range was 15.63 to 1000 ng/mL, with a sensitivity of 9.38 ng/mL and recovery of 80 to 120%; the manufacturer reports no significant cross-reactivity with structural analogs.

Serum vitamin E was measured using a double-antibody sandwich ELISA (Human Vitamin E ELISA Kit, Cat. No. MBS269047, MyBiosource Inc., San Diego, CA, USA) with a monoclonal capture antibody, a biotinylated detection antibody, horseradish peroxidase-avidin conjugation, and TMB detection at 450 nm. The analytical range was 1.56 to 100 µg/mL, with a sensitivity of 0.5 µg/mL, an intra-assay coefficient of variation ≤ 8%, and an inter-assay coefficient of variation ≤12%. This kit quantifies total circulating vitamin E without manufacturer-specified selectivity for individual tocopherol forms, whereas the CALIPER reference intervals were established for α-tocopherol measured by HPLC [12]. Because α-tocopherol represents approximately 90% of circulating vitamin E in humans [19], a low total vitamin E value necessarily implies low α-tocopherol; any small overestimation from other tocopherol forms would, if present, lead to underestimation rather than overestimation of the true prevalence of deficiency, which is a conservative bias for the interpretation of the present data. The immunoassay platform used here, and the HPLC platform used for CALIPER are not directly interchangeable; therefore, the CALIPER cutoffs were used as an interpretive framework rather than as a validation reference. Accordingly, the measured variable is reported throughout as total vitamin E, and the absolute, E:C and E:TG classifications are exploratory applications of CALIPER-derived cutoffs to a different analytical method.

Retinol (ng/mL) was converted to µmol/L by dividing by 286.45, the molecular weight of all-trans-retinol. Vitamin E (µg/mL) was converted to µmol/L using the formula µg/mL × 1000 ÷ 430.71, based on the molecular weight of α-tocopherol. Total cholesterol and triglycerides (mg/dL) were converted to mmol/L using conversion factors of 38.67 and 88.57, respectively.

### 2.4. Classification of Vitamin Status

Vitamin A was classified using the WHO threshold for serum retinol (below 0.70 µmol/L) and the age-stratified CALIPER lower limits (below 0.30 µmol/L for ages 0 to 1 year, below 1.00 for ages 1 to 11 years, below 0.90 for ages 11 to 16 years, and below 1.00 for ages 16 to 19 years) [12,14].

Vitamin E status was assessed using three classifications: (i) absolute total vitamin E below age-stratified thresholds (below 5 µmol/L for those under 12 months, below 12 µmol/L for older children); (ii) E:C ratio, calculated as total vitamin E (µmol/L) divided by total cholesterol (mmol/L), with the CALIPER lower reference limit of 3.7; (iii) E:TG ratio, calculated as total vitamin E (µmol/L) divided by triglycerides (mmol/L), with the CALIPER lower reference limit of 8.5. The E:C and E:TG cutoffs derived from CALIPER apply to children aged 1 to 19 years; no CALIPER-derived lipid-adjusted cutoff is available for infants under 12 months of age. The four infants under 12 months in our cohort were included in the E:C and E:TG analyses using the same cutoffs. This extrapolation is exploratory and is flagged as such wherever these children affect a result: no lipid-adjusted cutoff has been derived for this age group, and infant lipid metabolism differs from that of older children, so the lipid-adjusted classification of these four children is not a reference-interval-based determination. They were retained in the primary analysis so that the reported cohort is the cohort seen, and the pre-specified sensitivity analysis in Section Sensitivity Analysis Excluding Infants Under 12 Months repeats every principal result with the four infants excluded. Because measurement was based on immunoassay rather than HPLC, the terminology “vitamin E inadequacy” or “low lipid-adjusted vitamin E index” is used throughout, reserving the term “deficiency” for classifications derived from HPLC-based measurement. Blood samples in this cohort were obtained after fasting, whereas the CALIPER reference intervals were established from non-fasting samples; the implications of this methodological difference are addressed in the Discussion.

### 2.5. Statistical Analysis

**Figure 2:**
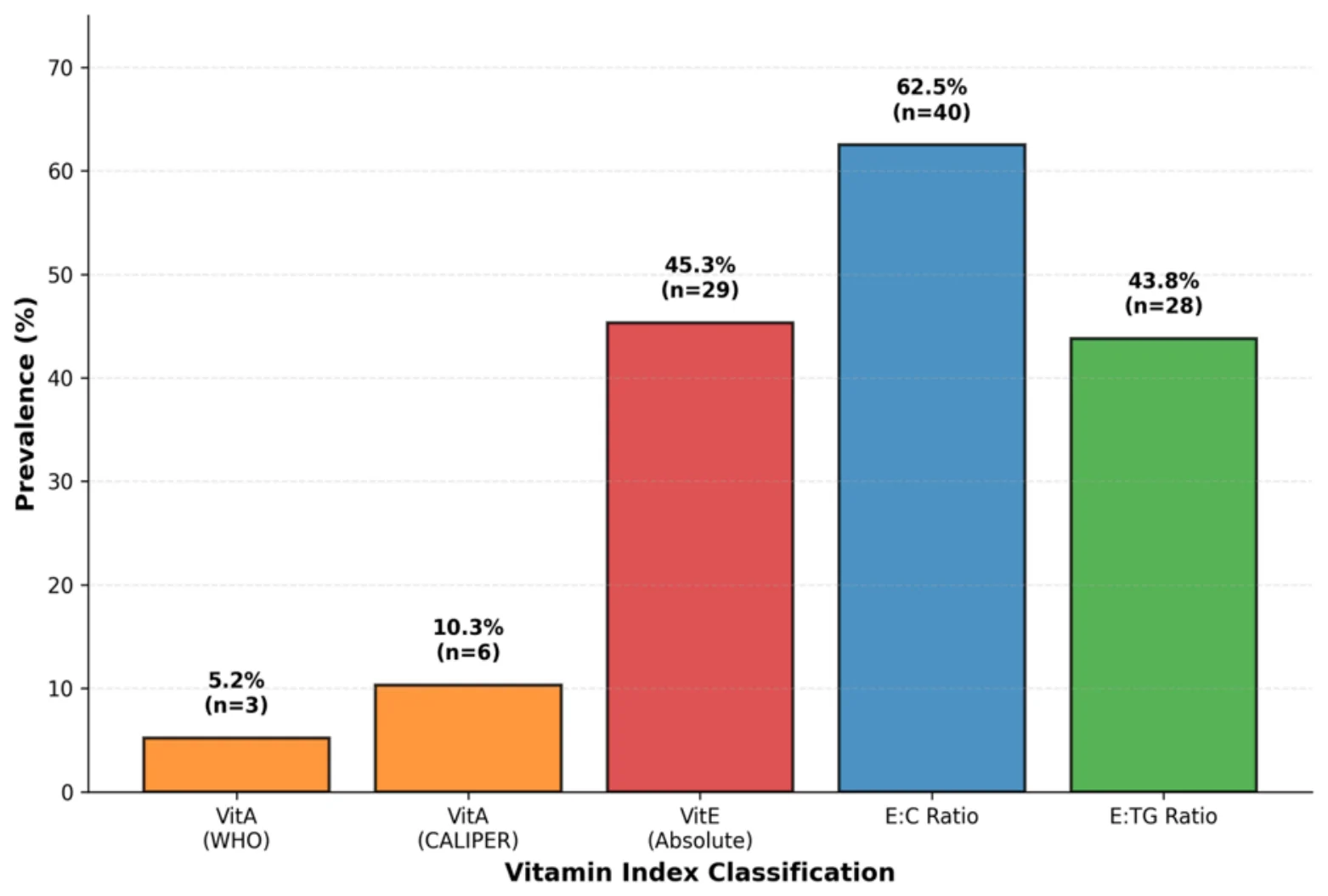
Prevalence of Low Vitamin Indices by Classification Criterion. Vitamin A status is largely preserved (5.2% below WHO threshold), whereas low vitamin E indices are common when assessed by lipid-adjusted ratios (E:C 62.5%, E:TG 43.8%) compared with absolute tocopherol deficiency (45.3%).

**Figure 3:**
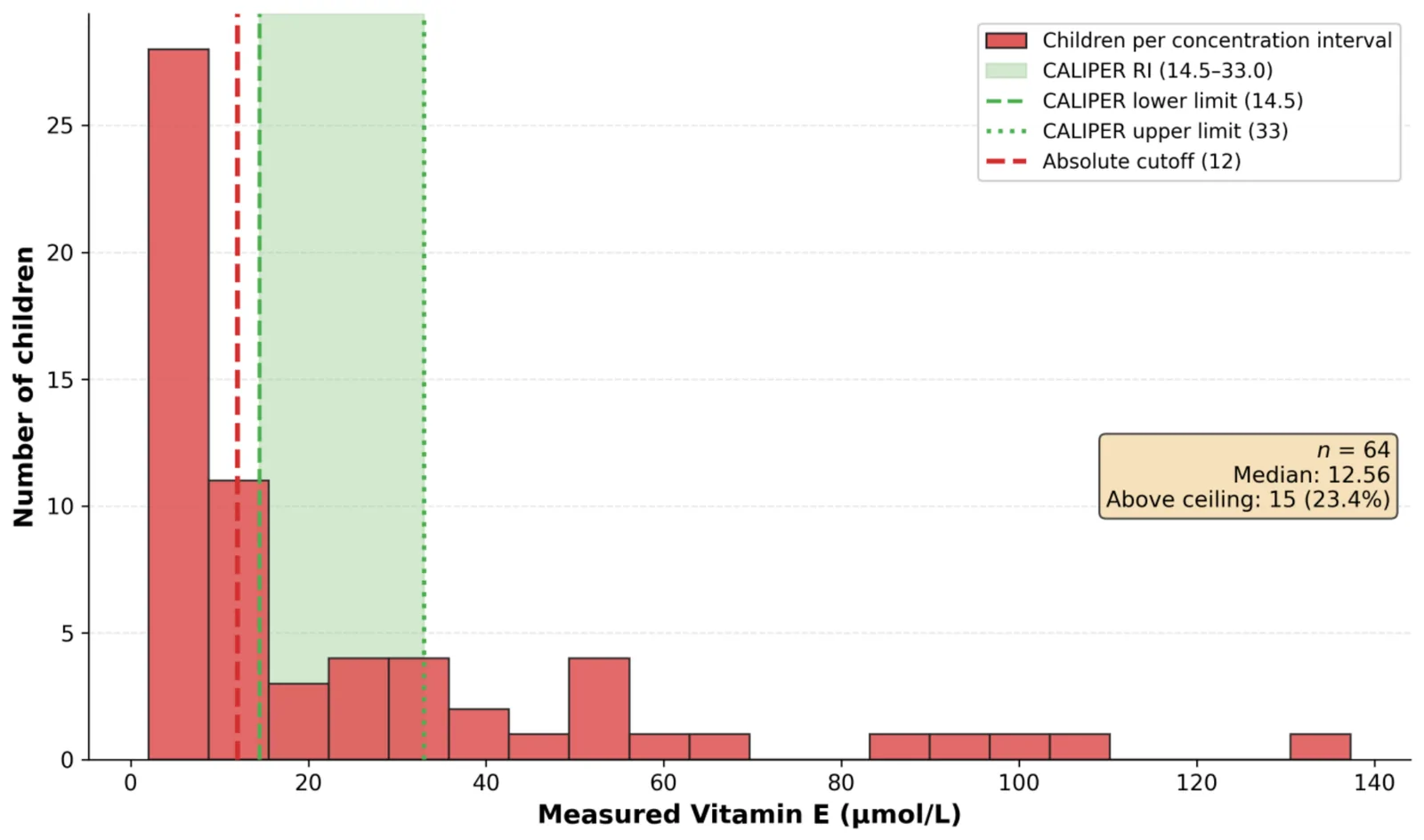
Distribution of Measured Serum Vitamin E. Red bars show the number of children in each concentration interval. The shaded band marks the CALIPER reference interval (14.5 to 33.0 µmol/L); the dashed red line indicates the absolute vitamin E cutoff (12 µmol/L); a substantial proportion of measured values fall above the reference ceiling, supporting the study’s exploratory framing.

**Figure 4:**
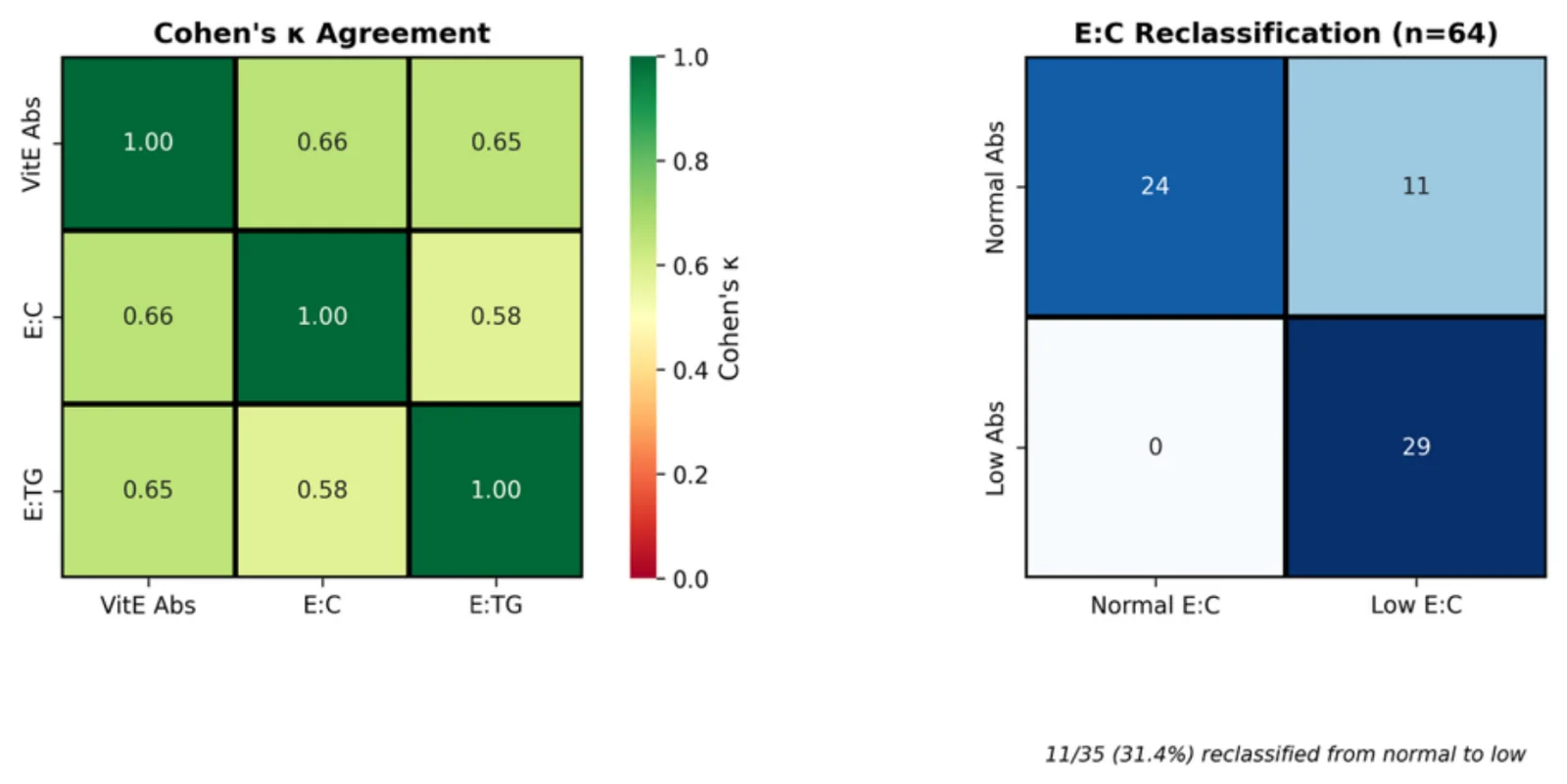
Agreement Between Vitamin E Classifications. Left panel: pairwise Cohen’s kappa coefficients show moderate to strong agreement between classifications (kappa = 0.58–0.66). Right panel: reclassification matrix showing that 11 of 35 children (31.4%) with normal absolute vitamin E are reclassified as having a low E:C ratio.

**Figure 5:**
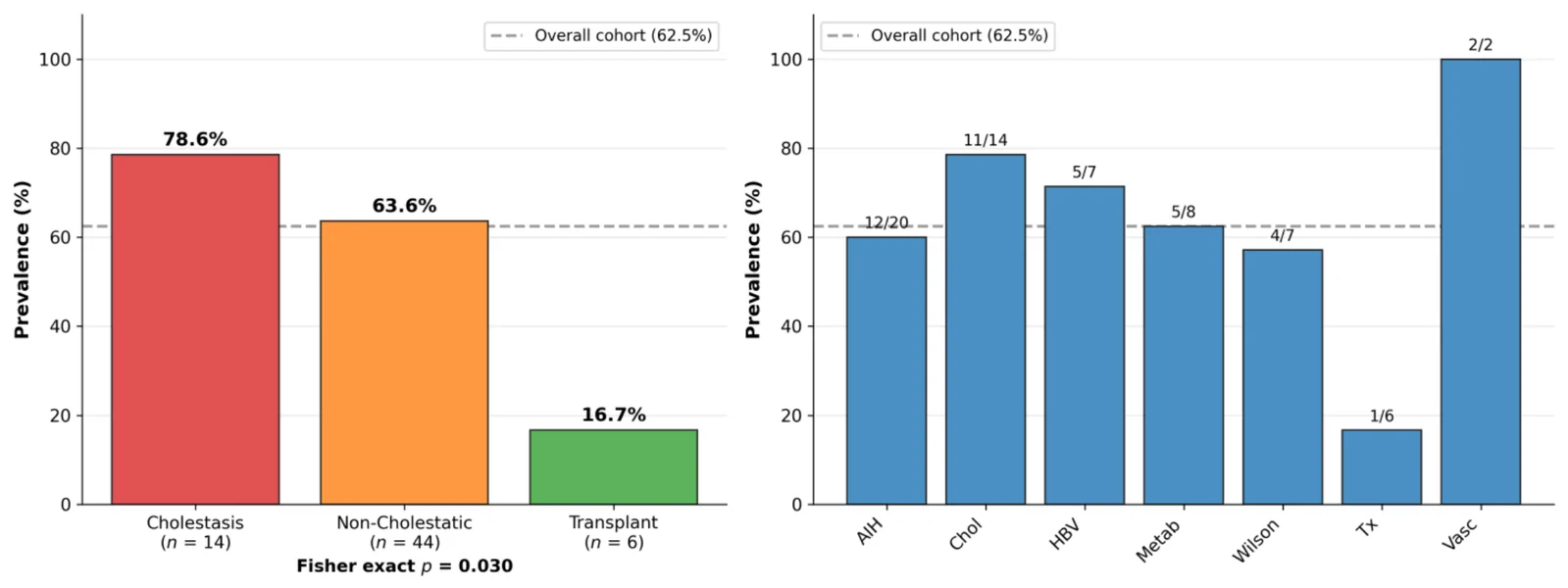
Prevalence of Low E:C Ratio Across Clinical Groups and Etiology. Left panel: categorical prevalence of low E:C (<3.7) differs significantly across three pre-specified clinical groups (cholestatic 78.6%, non-cholestatic 63.6%, post-transplant 16.7%; *p* = 0.030). Right panel: prevalence stratified by aetiological category.

**Figure 6:**
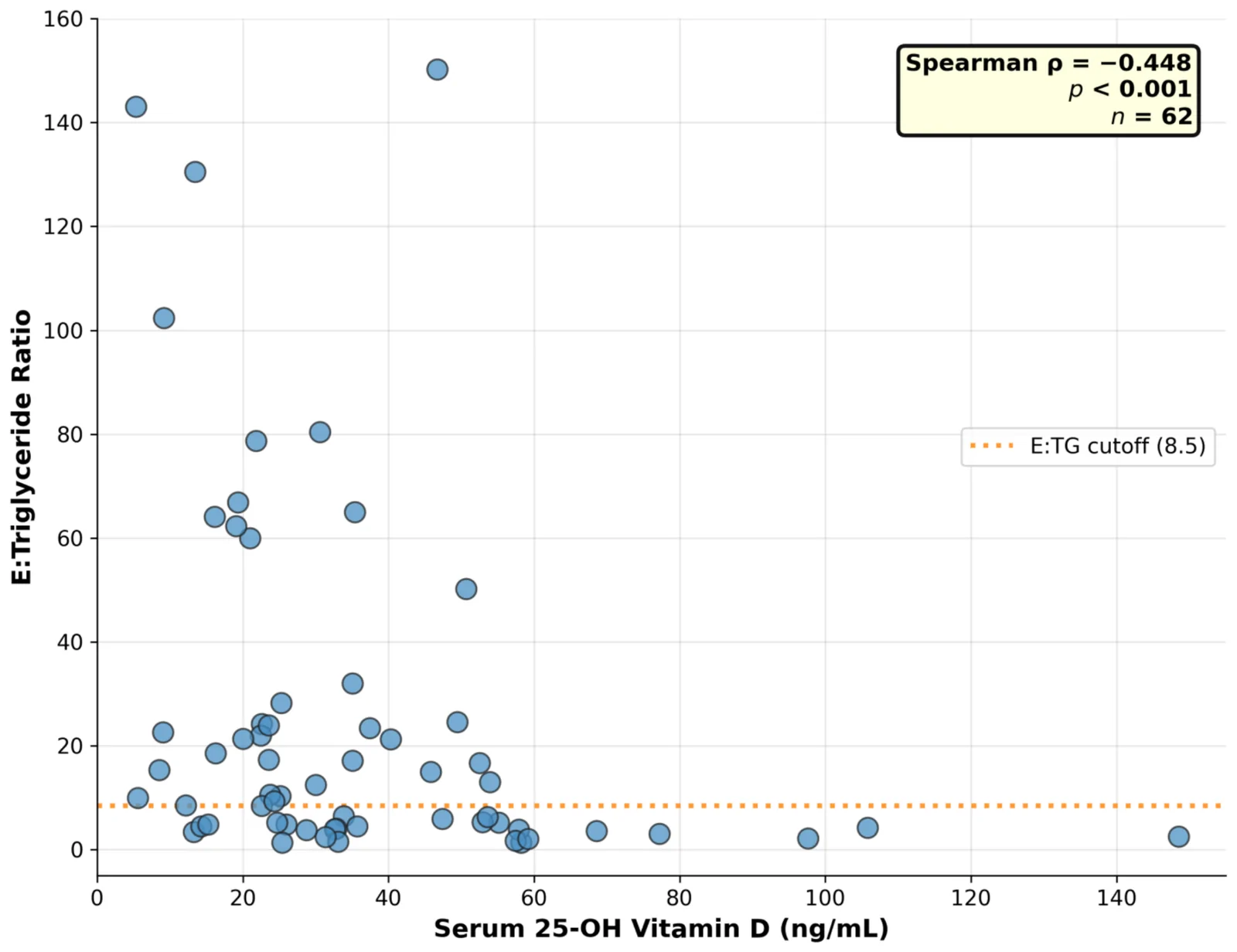
E:TG Ratio Versus Serum 25-Hydroxyvitamin D. Inverse correlation (Spearman rho = −0.45, *p* < 0.001, *n* = 62) is consistent with triglyceride-mediated confounding of the E:TG ratio and age-related confounding. Children with lower 25(OH)D had, on average, a higher E:TG index—a direction contrary to joint fat-soluble vitamin depletion and consistent with triglyceride-mediated confounding of the ratio and dual bile-flow-dependent effects in cholestasis (see Section 4).

Statistical analyses were conducted in jamovi 2.7.30 (jamovi project; jamovi.org), which runs on R 4.5. Because all vitamin variables deviated from normality (Shapiro–Wilk *p* < 0.001), continuous variables were compared with rank-based tests (Kruskal–Wallis across three groups, Mann–Whitney U between two) and associations between continuous variables were assessed with Spearman rank correlation. Categorical variables were compared with Fisher exact test, which was used in preference to the chi-square test because expected cell counts below five arise in every comparison involving the six-child post-transplant group. No formal correction for multiple comparisons was applied: the analyses are exploratory, so *p*-values are reported as descriptive quantities alongside effect estimates and confidence intervals rather than as confirmatory tests. Publication-ready figures were generated in Google Colaboratory (Google LLC, Mountain View, CA, USA; https://colab.research.google.com, accessed on 1 October 2026) using Python 3.9 (matplotlib 3.5, seaborn 0.12, scipy 1.9) for Figures 1, 2 and 4, and Python 3.13 (matplotlib 3.10, scipy 1.16) for Figures 3, 5 and 6, which were regenerated at the proof stage, at 300 dpi.

During the preparation of this manuscript, the authors used Claude (Anthropic) to assist with synthesizing published literature and with improving the language and structure of the text. The tool was not used for study design, patient screening, data collection, statistical analysis, or the generation of data, results, or conclusions. The authors reviewed and edited all output and take full responsibility for the content.

## 3. Results

### 3.1. Cohort Characteristics

The analytic cohort comprised 66 children with a median age of 8.5 years (IQR 4.3 to 11.8; range 4 months to 17.9 years). The underlying liver diseases included autoimmune hepatitis (21 children, 31.8%), cholestatic diseases (including biliary atresia and genetic cholestatic disorders) (15, 22.7%), inherited metabolic liver diseases (8, 12.1%), Wilson disease (7, 10.6%), chronic hepatitis B (7, 10.6%), and vascular disease (2, 3.0%). Six children (9.1%) had previously undergone liver transplantation. For the pre-specified secondary analysis, 15 children (22.7%) had biochemical cholestasis, 45 (68.2%) had non-cholestatic CLD, and 6 (9.1%) were post-transplant. The cohort was predominantly compensated: 47 children (71.2%) were staged F0 to F1, 19 (28.8%) had significant fibrosis (F ≥ 2), and 9 (13.6%) had cirrhosis (F4). Baseline characteristics are summarized in Table 1.

### 3.2. Vitamin A Status

Vitamin A was measurable in 58 children (median 1.73 µmol/L; IQR 1.57 to 1.96; range 0.42 to 4.69). Three children (5.2%) were below the WHO threshold of 0.70 µmol/L, and 6 (10.3%) were below the age-stratified CALIPER lower limit (Figure 2). No child had clinical features of vitamin A deficiency at the study visit. Vitamin A did not correlate with any biochemical marker of hepatic function, fibrosis stage, or age (all *p* > 0.6). Vitamin A did not differ across the three clinical groups (Kruskal–Wallis *p* = 0.55) or across fibrosis stages (*p* = 0.41). All three children below the WHO threshold and all six below the CALIPER limit were in non-cholestatic groups (autoimmune hepatitis, metabolic, and vascular disease), whereas retinol was preserved in the cholestatic and post-transplant groups. This pattern is compatible with suppression of RBP4 synthesis during active hepatocellular inflammation, independent of fat malabsorption [13].

### 3.3. Vitamin E and Lipid-Adjusted Indices

Vitamin E was measurable in 64 children (median 12.56 µmol/L; IQR 3.23 to 31.39). The observed range was wide (2.00 to 137.31 µmol/L), and 15 children (23.4%) had values above the CALIPER upper limit of 33 µmol/L, with 5 (7.8%) above 66 µmol/L (Figure 3). These values exceed the ceiling reported in HPLC-based pediatric reference intervals. Without a parallel HPLC measurement, this study cannot establish how far they reflect true lipid-related variation and how far they reflect the behaviour of the immunoassay at the extremes; both are plausible and neither is demonstrated here. This distribution motivated the study’s exploratory framing: interpretation was based on classification patterns rather than on precise quantification of absolute concentrations.

By absolute concentration, 29 of 64 children (45.3%) had a low vitamin E concentration. The E:C ratio was low in 40 of 64 (62.5%), and the E:TG ratio in 28 of 64 (43.8%; Figure 2, Table 2). Absolute vitamin E correlated positively with triglycerides (rho = +0.30, *p* = 0.015) but not with total cholesterol (rho = +0.09, *p* = 0.47), consistent with the lipoprotein dependence of circulating vitamin E and supporting the rationale for lipid-adjusted interpretation.

### 3.4. Reclassification Between Vitamin E Classifications

Because the three vitamin E classifications capture different aspects of the same underlying variable, we quantified how often they classified individual children differently. Complete data for all three classifications were available for 64 children. Agreement between absolute vitamin E and the E:C ratio was moderate (kappa = 0.66); agreement between absolute vitamin E and the E:TG ratio was similar (kappa = 0.65); and agreement between the two lipid-adjusted ratios was moderate (kappa = 0.58). Among 35 of 64 children with a normal absolute vitamin E concentration, 11 (31.4%) were reclassified as having a low E:C ratio, and 5 (14.3%) as having a low E:TG ratio (Table 3, Figure 4). Conversely, 6 children with a low absolute vitamin E concentration had a normal E:TG ratio, while none with a low absolute concentration were reclassified as having a normal E:C ratio. The reclassification is therefore unidirectional for the E:C ratio: it identifies children whose absolute value would have suggested adequate status, but whose lipid-adjusted index is low. This pattern is consistent with the mechanism first described by Sokol et al., in whose series 27% of children with biopsy-confirmed vitamin E deficiency had normal absolute α-tocopherol [7]. In our cohort, the E:C ratio was the classification that most frequently changed clinical interpretation.

### 3.5. Secondary Analysis Across Three Clinical Groups

The pre-specified three-group analysis compared biochemical cholestasis (*n* = 15), non-cholestatic CLD (*n* = 45), and post-transplant recipients (*n* = 6). Vitamin A did not differ across groups (medians 1.78, 1.71, and 1.64 µmol/L; Kruskal–Wallis *p* = 0.55). Absolute vitamin E, E:C, and E:TG did not differ significantly across the three groups on the continuous scale (Kruskal–Wallis *p* = 0.26, 0.11, and 0.25), reflecting the small transplant subgroup and the wide within-group dispersion. However, the categorical prevalence of a low E:C ratio differed markedly (Table 4, Figure 5): 11 of 14 cholestatic children (78.6%), 28 of 44 non-cholestatic children (63.6%), and 1 of 6 post-transplant children (16.7%; Fisher exact *p* = 0.030). The E:TG ratio showed a similar gradient (57.1%, 43.2%, and 16.7%).

#### Sensitivity Analysis Excluding Infants Under 12 Months

Because pediatric lipid metabolism differs in the first year of life, a pre-specified sensitivity analysis was performed excluding the 4 infants under 12 months of age (two in the cholestatic group and two in the post-transplant group). The main conclusions were preserved. The categorical difference in low E:C prevalence across the three clinical groups remained statistically significant (cholestatic: 9 of 12, 75.0%; non-cholestatic: 28 of 44, 63.6%; post-transplant: 0 of 4, 0%; Fisher exact *p* = 0.025). No post-transplant child had a low E:C ratio in this restricted analysis. Because no child was measured both before and after transplantation, this is a difference between groups of different children and not a change observed within any child. The reclassification pattern was essentially unchanged: 10 of 33 children (30.3%) with a normal absolute vitamin E concentration were reclassified as having a low E:C ratio (kappa 0.67), compared with 11 of 35 (31.4%; kappa 0.66) in the full cohort. The E:TG ratio remained inversely correlated with 25(OH)D (Spearman rho = −0.39; *p* = 0.002; *n* = 58). The concordance of these findings with the primary analysis argues that the reported results are not driven by the small infant subgroup and are robust to age as a potential confounder.

### 3.6. Correlations with Biochemical Markers and Vitamin D Status

Serum 25-hydroxyvitamin D was measurable in 64 children (median 31.0 ng/mL; IQR 21.6 to 49.7). Using conventional pediatric thresholds, 14 children (21.9%) were vitamin D deficient (<20 ng/mL), 16 (25.0%) were insufficient (20 to 30 ng/mL), and 34 (53.1%) had sufficient values (≥30 ng/mL); thus, approximately half of the cohort had suboptimal vitamin D status. Correlations between vitamin indices and biochemical markers are summarized in Table 5. An inverse correlation was observed between the E:TG ratio and serum 25(OH)D (Spearman rho = −0.45, *p* < 0.001, *n* = 62; Figure 6). Within the same cohort, 25(OH)D was positively correlated with serum triglycerides (Spearman ρ = +0.36, *p* = 0.004, *n* = 64); the E:C ratio showed a substantially weaker inverse correlation with 25(OH)D (rho = −0.26, *p* = 0.041), and absolute vitamin E was not significantly correlated with 25(OH)D (rho = −0.23; Table 5). The mechanistic interpretation of these correlations is developed in the Discussion. Correlations between vitamin indices and GGT and bilirubin were in the expected direction but did not reach statistical significance. Vitamin A showed no significant biochemical correlation.

### 3.7. Vitamin Indices and Hepatic Fibrosis

We did not detect a difference in any vitamin A or vitamin E index between children with and without significant fibrosis (F ≥ 2) or across the five fibrosis stages (all Kruskal–Wallis *p* > 0.20; Table 6). The categorical prevalence of a low E:C ratio was numerically higher in cirrhotic children than in non-cirrhotic children (7 of 8, 87.5% versus 33 of 56, 58.9%; Fisher *p* = 0.24), but the difference was not statistically significant. The advanced-fibrosis and cirrhotic subgroups were small (14 and 9 children, respectively), so these comparisons were underpowered; the absence of a significant difference should not be interpreted as evidence that no relationship exists. Given the cross-sectional design, progression-related vitamin changes cannot be assessed.

## 4. Discussion

In this exploratory single-center study of 66 children with CLD of mixed etiology, four findings merit emphasis. First, lipid-adjusted interpretation of vitamin E reclassified a substantial proportion of children compared with the absolute vitamin E concentration: approximately one in three children whose absolute value appeared adequate had a low E:C ratio, and the reclassification was unidirectional for the E:C ratio, adding children to rather than removing them from the low-index category. Second, the post-transplant group had the lowest prevalence of a low E:C ratio of the three groups (Fisher exact *p* = 0.030), a difference resting on six children. Third, an inverse correlation was observed between the E:TG ratio and 25(OH)D (rho = −0.45; *p* < 0.001). Part of this follows mathematically from the construction of the ratio, since triglycerides are its denominator and were positively correlated with 25(OH)D in this cohort; any further physiological interpretation is hypothesis-generating and is presented as such below. Fourth, vitamin A status was largely preserved in this predominantly compensated cohort.

The reclassification pattern is consistent with the mechanism first demonstrated by Sokol et al. in 1984, in whose series 27% of children with biopsy-confirmed vitamin E deficiency had absolute serum α-tocopherol levels within the reference range [7]. The unidirectional nature of the reclassification supports the pathophysiological interpretation that hyperlipidemia in CLD elevates the serum α-tocopherol pool without a proportionate increase in tissue availability; the E:C ratio then correctly identifies these children as having an inadequate index. The moderate kappa agreement (0.58 to 0.66) among the three vitamin E classifications confirms that they are not interchangeable and supports parallel reporting.

An important methodological clarification concerns the choice of immunoassay over HPLC. The vitamin E ELISA test used in this study quantifies total circulating vitamin E, whereas the CALIPER reference intervals were established for α-tocopherol measured by HPLC [12]. Because α-tocopherol accounts for approximately 90% of circulating vitamin E in humans, a low total vitamin E value necessarily implies low α-tocopherol; any small overestimation of total vitamin E by other tocopherol forms (β, γ, δ) would lead to underestimating the true prevalence of low α-tocopherol status, a conservative bias for our conclusions [20,21,22]. One consideration is specific to the reclassification analysis: both classifications derive from the same vitamin E measurement in the same child, so a systematic bias of the immunoassay shifts both and cannot by itself produce the observed reclassification, which depends on the ratio to cholesterol. This argument concerns the reclassification pattern only; it does not extend to the prevalence estimates, which remain dependent on the platform. That said, the two platforms are not directly interchangeable, and the wide observed range of measured vitamin E in our cohort (2 to 137 µmol/L, with 23% above the CALIPER upper limit) reflects a combination of true lipid-related variation and immunoassay behavior at the extremes. For this reason, we frame the study as exploratory and interpret prevalence estimates as indicative rather than as validated diagnostic thresholds; the interpretive message concerns classification patterns and their clinical implications, not exact prevalence.

Across the three clinical groups, the categorical prevalence of a low E:C ratio increased from post-transplant (17%) to non-cholestatic CLD (64%) to cholestatic disease (79%), and this gradient was statistically significant (Fisher exact *p* = 0.030). The post-transplant group thus had the lowest prevalence of a low E:C ratio, a signal resting on six children. One interpretation, which this design cannot test, is that restoration of bile flow after transplantation is followed by recovery of vitamin E status; because the comparison is between different children rather than within the same child over time, the data establish an association with post-transplant status and not a change produced by transplantation. At the pre-transplant end of the spectrum, the difference between cholestatic and non-cholestatic groups in this cohort may partly reflect the cholestatic group’s younger age, which the sample size did not allow us to adjust for.

The inverse correlation between the E:TG ratio and serum 25(OH)D is contrary to the direction expected if fat-soluble vitamin depletion co-clustered in this cohort: under joint depletion, both low 25(OH)D and low E:TG would occur together, producing a positive correlation. Instead, children with lower 25(OH)D had a higher (more adequate-appearing) E:TG index.

Two non-mutually exclusive mechanisms are consistent with this pattern. First, in this cohort, 25(OH)D was positively correlated with serum triglycerides (Spearman ρ = +0.36, *p* = 0.004, *n* = 64). Because triglycerides are the denominator of the E:TG ratio, higher 25(OH)D tracked with higher triglycerides and, mechanically, produced lower E:TG values, accounting for the observed inverse 25(OH)D–E:TG relationship. This positive intra-cohort 25(OH)D–TG correlation is contrary to the inverse relationship typically reported in general pediatric populations and may reflect the effect of concurrent clinical management (e.g., vitamin D supplementation) or the specific aetiological composition of the cohort; either way, its mathematical consequence for the E:TG denominator is direct. Second, in the cholestatic subgroup, reduced bile flow simultaneously elevates triglycerides (lowering the E:TG denominator) and impairs vitamin D absorption; if 25(OH)D is partly restored by routine clinical supplementation, this dual bile-flow-dependent dysregulation can generate an inverse rather than a positive 25(OH)D–E:TG relationship. Two internal observations support this interpretation: (i) the E:C ratio, which does not carry triglycerides in its denominator, showed a much weaker inverse correlation with 25(OH)D (rho = −0.26; *p* = 0.041), and (ii) vitamin E absolute concentration was not significantly correlated with 25(OH)D.

This finding does not undermine the reclassification and transplant results, which rely on the E:C ratio; however, it suggests that the E:TG ratio is sensitive to non-CLD determinants of triglycerides and is less suitable as a stand-alone index in a mixed-etiology pediatric cohort. In this exploratory cohort, the E:C ratio showed greater potential than the E:TG ratio for lipid-adjusted interpretation of vitamin E status. Prospective validation against HPLC-based measurements and established functional indices is required before its routine clinical adoption. Because these analyses were exploratory, no finding can be considered confirmed on the basis of this cohort. Of the four principal tests, only the E:TG–25(OH)D correlation (rho = −0.45, *p* < 0.001) would remain significant under a Bonferroni-corrected threshold (α = 0.0125); the three-group gradient in low E:C prevalence (Fisher exact *p* = 0.030) would not. We interpret the E:TG–25(OH)D correlation as reflecting triglyceride- and cholestasis-related confounding of the ratio rather than fat-soluble vitamin depletion, so its statistical strength does not by itself identify a clinical signal to pursue. The reclassification captured by the E:C ratio and the lower prevalence of a low E:C ratio in the post-transplant subgroup are the associations of clinical interest; all of these findings would require independent validation in a separate cohort.

The low prevalence of vitamin A deficiency in our cohort (5.2% by the WHO threshold, 10.3% by CALIPER) contrasts with the 67–77% reported in predominantly cholestatic pediatric series. Two explanations are likely. First, serum retinol is homeostatically buffered and remains within range until hepatic stores are markedly depleted, so absolute serum retinol underestimates depletion; functional indices such as the retinol: RBP4 ratio or the relative dose–response would be expected to reclassify some apparently sufficient children. Second, our cohort was dominated by non-cholestatic disease, and the few children below the WHO threshold belonged to non-cholestatic groups (autoimmune hepatitis and metabolic disease). This pattern is compatible with reduced hepatic synthesis of RBP4 during active inflammation, as RBP4 is a negative acute-phase reactant independent of malabsorption [13].

The absence of any relationship between vitamin indices and fibrosis stage merits comment. Our cohort was predominantly without significant fibrosis (71% F0 to F1), and the advanced-fibrosis and cirrhotic subgroups were small (14 and 9 children), so the null result reflects limited statistical power rather than definitive evidence of no association. That said, the observation that a low E:C ratio is prevalent even in F0 to F1 (approximately 60%) is consistent with vitamin E status being driven primarily by bile flow rather than by fibrotic remodeling.

It is worth contrasting the pediatric picture with the adult literature. In adults with advanced CLD, vitamin A deficiency is highly prevalent and tracks disease severity and portal hypertension, as recently confirmed in a large series of adults with cirrhosis [23]. The adult vitamin E literature is more heterogeneous: Sokol et al. documented low serum α-tocopherol and biopsy-confirmed vitamin E deficiency in a series of 60 adults with chronic cholestatic disease [24], but the epidemiology of vitamin E deficiency in adult mixed-etiology CLD is less well characterized than in the pediatric cholestatic population. Our series shows a pediatric-specific pattern: preserved vitamin A and a frequently low vitamin E index. The most plausible explanations are the cohort’s younger age, the predominance of non-cirrhotic cases, and the etiological composition. Fat-soluble vitamin surveillance in pediatric CLD should therefore not be extrapolated directly from adult recommendations, and existing pediatric guidance from ESPGHAN and NASPGHAN emphasizes regular monitoring and supplementation of fat-soluble vitamins in children with cholestatic and post-transplant CLD [25]; these recommendations are reaffirmed in the recent Australasian (AuSPEN) consensus on nutrition support in paediatric CLD [26].

### 4.1. Limitations

Several limitations apply. First, and most importantly, retinol and vitamin E were measured by ELISA rather than by HPLC. The two platforms are not directly interchangeable, and the CALIPER reference intervals were established by HPLC. Although the direction of any bias from the total vitamin E vs. α-tocopherol issue would be conservative (see Section 4), the wide distribution of measured values reflects a combination of true lipid-related variation and immunoassay behavior at the extremes. For this reason, the study is framed as exploratory, and prevalence estimates should be regarded as descriptive rather than as validated diagnostic thresholds. A direct HPLC comparison in a subset is warranted.

Second, blood samples were obtained in a fasting state, whereas the CALIPER reference intervals were established using non-fasting samples [12]. Triglycerides are most affected by prandial status, with fasting values systematically lower than non-fasting values [27]. The E:TG ratio in our fasting cohort may therefore be systematically higher than in the CALIPER reference population, which could lead to underestimating the proportion of children below the E:TG cutoff. The E:C ratio is less affected because total cholesterol shows minimal prandial variation; this may partly explain why the E:C ratio identified more children with a low index (62.5%) than the E:TG ratio (43.8%).

Another limitation is that the study is cross-sectional and cannot assess changes over time, response to supplementation, or clinical outcomes. The design also does not fix the direction of any association: vitamin E indices and markers of liver disease were measured at the same visit, so reverse causation, in which disease state follows rather than precedes vitamin status, cannot be excluded. We did not measure RBP4 and therefore could not calculate the retinol: RBP4 ratio, the functional index recommended in CLD [13]. Also, the phospholipid-based Sokol vitamin E to total lipid ratio, considered the gold standard for cholestatic patients by ESPGHAN/NASPGHAN guidelines [25], could not be calculated because phospholipid measurements were unavailable; the E:C and E:TG ratios from CALIPER were used as accessible alternatives. Subgroup sizes for post-transplant (*n* = 6), vascular disease (*n* = 2), and advanced fibrosis (F ≥ 3, *n* = 14) were small. Two further limitations apply specifically to the transplant comparison: time since transplantation, and immunosuppressive and supplementation regimens were not recorded, so the observed difference cannot be attributed to transplantation rather than to other characteristics of that group. This is a single-center cohort, and generalizability to other populations is limited. Dietary intake and supplementation history were not recorded, which may confound the relationship between clinical group and vitamin status. Finally, because most analyses were exploratory, no formal multiple-testing correction was applied; the reported *p*-values should be interpreted accordingly.

### 4.2. Clinical and Research Implications

Within these constraints, the findings support jointly evaluating the absolute vitamin E concentration and lipid-adjusted indices when assessing vitamin E status in children with CLD, rather than relying on absolute concentration alone. The E:C ratio is simple and readily available from a routine fasting lipid panel, and in our cohort, it identified the largest proportion of children with a low index. In practical terms, the 11 children whose absolute vitamin E concentration suggested adequacy but whose E:C ratio was low are the children a lipid-adjusted index identifies, and an absolute concentration does not. Whether they benefit from closer monitoring or from supplementation is a question for future investigation: this study did not validate the E:C cutoff against HPLC or against any clinical endpoint, so it cannot support a monitoring recommendation. This is consistent with the ESPGHAN/NASPGHAN recommendation that vitamin E surveillance in pediatric cholestatic CLD uses lipid-adjusted indices rather than absolute concentrations. The lower prevalence of a low E:C ratio among post-transplant participants is consistent with the clinical expectation that restoration of bile flow is followed by recovery of fat-soluble vitamin absorption, although six children cannot establish it. The prevalence of a low index in the non-cholestatic group (64%) argues against assuming that a non-cholestatic etiology confers protection. The observation that approximately half of the cohort had suboptimal vitamin D status supports monitoring vitamin D as part of routine fat-soluble vitamin surveillance in pediatric CLD [28], independently of the methodological E:TG–25(OH)D pattern discussed above.

### 4.3. Future Perspectives

Four lines of work follow from these results. First, a direct comparison with HPLC-based measurement on a subset of the same samples would establish how far the immunoassay values and the classifications derived from them correspond to α-tocopherol as CALIPER defines it; this is the step on which the interpretation of every prevalence reported here depends. Second, prospective studies with documented supplementation and dietary intake, functional vitamin A testing including the retinol to RBP4 ratio, and larger etiologically homogeneous subgroups are needed to translate these exploratory findings into confirmed clinical thresholds. Third, larger cohorts enriched in advanced disease are needed to test whether fat-soluble vitamin inadequacy tracks with fibrosis progression, which the present sample was underpowered to address. Fourth, whether combined vitamin D and vitamin E supplementation strategies confer additional benefit will require prospective evaluation, as will the question of whether children identified only by a lipid-adjusted index derive clinical benefit from that identification.

## 5. Conclusions

In this exploratory single-center pediatric CLD cohort assessed by immunoassay, vitamin A status was largely preserved, whereas lipid-adjusted vitamin E indices reclassified a substantial proportion of children compared with the absolute vitamin E concentration. Across the three clinical groups, the lowest prevalence of a low E:C ratio was observed among the six post-transplant participants (Fisher exact *p* = 0.030); no child was studied across transplantation, so this is a difference between groups and not an observed change. An inverse correlation between the E:TG ratio and 25(OH)D, opposite to that expected under joint fat-soluble vitamin depletion, is most consistent with triglyceride-mediated confounding and cholestasis-driven dysregulation of lipid handling and vitamin D absorption, and is one reason the E:C ratio, whose denominator is not triglycerides, behaved more consistently than the E:TG ratio in this cohort. Because measurement was based on immunoassay rather than HPLC, the key message is that adding a routine lipid panel to the vitamin E result changes clinical classification and identifies children who would be missed by the absolute vitamin E concentration alone; confirmation with HPLC-based measurement in larger prospective studies is warranted. The terms “vitamin E inadequacy” or “low lipid-adjusted index” are preferred over “deficiency” when interpretation relies on immunoassay-based measurement.

## Figures and Tables

**Table 1:** Demographic, clinical, and biochemical characteristics of the study cohort (*n* = 66).

Characteristic		Values
Age (years)	Median (IQR)	8.5 (4.3–11.8)
Age < 12 months	*n* (%)	4 (6.1)
Age 1–11 years	*n* (%)	40 (60.6)
Age 11–16 years	*n* (%)	20 (30.3)
Age 16–18 years	*n* (%)	2 (3.0)
Primary diagnosis		
Autoimmune hepatitis	*n* (%)	21 (31.8)
Cholestatic disease	*n* (%)	15 (22.7)
Inherited metabolic liver diseases	*n* (%)	8 (12.1)
Wilson disease	*n* (%)	7 (10.6)
Chronic hepatitis B	*n* (%)	7 (10.6)
Vascular disease	*n* (%)	2 (3.0)
Post-liver transplant	*n* (%)	6 (9.1)
Secondary analysis group		
Biochemical cholestasis	*n* (%)	15 (22.7)
Non-cholestatic CLD	*n* (%)	45 (68.2)
Post-liver transplant	*n* (%)	6 (9.1)
Fibrosis stage		
F0	*n* (%)	35 (53.0)
F1	*n* (%)	12 (18.2)
F2	*n* (%)	5 (7.6)
F3	*n* (%)	5 (7.6)
F4 (cirrhosis)	*n* (%)	9 (13.6)
Laboratory parameters		
GGT (U/L)	Median (IQR)	30.8 (18.1–97.8)
Total bilirubin (mg/dL)	Median (IQR)	0.53 (0.43–0.88)
Direct bilirubin (mg/dL)	Median (IQR)	0.18 (0.14–0.43)
Cholinesterase (U/L)	Median (IQR)	8745 (5813–9773)
Total cholesterol (mmol/L)	Median (IQR)	4.24 (3.76–4.96)
Triglycerides (mmol/L)	Median (IQR)	0.89 (0.60–1.40)
25(OH)D (ng/mL)	Median (IQR)	31.0 (21.6–49.7)

Abbreviations: CLD, chronic liver disease; GGT, gamma-glutamyltransferase; IQR, interquartile range.

**Table 2:** Prevalence of low vitamin index by classification criterion.

Criterion	Cutoff	Low/Assessed	Prevalence (%)
Vitamin A, WHO	<0.70 µmol/L	3/58	5.2
Vitamin A, CALIPER (age)	age-stratified	6/58	10.3
Vitamin E, absolute (age)	<5 or <12 µmol/L	29/64	45.3
Vitamin E, E:C ratio	<3.7	40/64	62.5
Vitamin E, E:TG ratio	<8.5	28/64	43.8

CALIPER lower reference limits were used for the age-stratified vitamin A cutoffs and for the E:C and E:TG ratios [12]. WHO, World Health Organization.

**Table 3:** Cross-classification between absolute and lipid-adjusted vitamin E indices (*n* = 64).

Vitamin E/E:C Ratio	E:C Ratio ≥3.7	E:C Ratio <3.7	E:TG Ratio ≥8.5	E:TG Ratio <8.5
Vitamin E absolute normal (*n* = 35)	24	11	30	5
Vitamin E absolute low (*n* = 29)	0	29	6	23
Cohen’s kappa vs. absolute		0.66		0.65

**Table 4:** Vitamin A and vitamin E indices across three clinical groups.

Variable	Cholestatic (*n* = 15)	Non-Cholestatic (*n* = 45)	Post-Transplant (*n* = 6)	*p*
Vitamin A, µmol/L, median (IQR)	1.78 (1.59–3.26)	1.71 (1.54–1.93)	1.64 (1.54–3.43)	0.55
Vitamin A below WHO, *n* (%)	0/13 (0)	3/39 (7.7)	0/6 (0)	n/a
Vitamin A below CALIPER, *n* (%)	0/13 (0)	6/39 (15.4)	0/6 (0)	n/a
Vitamin E, µmol/L, median (IQR)	10.15 (3.47–16.40)	12.15 (3.13–31.96)	37.33 (17.55–53.10)	0.26
E:C ratio, median (IQR)	2.13 (0.68–3.34)	2.53 (0.79–8.62)	8.11 (7.60–10.02)	0.11
E:TG ratio, median (IQR)	5.80 (2.62–10.13)	10.13 (2.94–24.42)	22.69 (18.42–31.51)	0.25
Vitamin E absolute low, *n* (%)	6/14 (42.9)	22/44 (50.0)	1/6 (16.7)	0.36
E:C < 3.7, *n* (%)	11/14 (78.6)	28/44 (63.6)	1/6 (16.7)	0.030
E:TG < 8.5, *n* (%)	8/14 (57.1)	19/44 (43.2)	1/6 (16.7)	0.29

*p*-values from the Kruskal–Wallis test for continuous variables and the Fisher exact test for categorical variables. Denominators reflect available measurements. IQR, interquartile range.

**Table 5:** Spearman correlations between vitamin indices and biochemical/clinical markers.

Clinical and Biochemical Parameters	Vitamin A	Vitamin E (Absolute)	E:C Ratio	E:TG Ratio
Age (months)	−0.03	+0.21	+0.11	+0.19
GGT	−0.05	+0.02	−0.09	−0.23
Total bilirubin	−0.01	−0.11	−0.21	−0.12
Direct bilirubin	−0.02	n/a	−0.17	−0.06
Cholinesterase	−0.04	n/a	n/a	n/a
Total cholesterol	n/a	+0.09	n/a	n/a
Triglycerides	n/a	+0.30 *	n/a	−0.24
25(OH)D	−0.04	−0.23	−0.26 *	−0.45 **
Fibrosis (F stage)	+0.04	n/a	−0.01	+0.05

Values are Spearman rank correlation coefficients (rho). * *p* < 0.05; ** *p* < 0.001. GGT, gamma-glutamyltransferase. Fields marked n/a were not analyzed for that pair.

**Table 6:** Vitamin indices by fibrosis stage.

Fibrosis Stage	F0 (*n* = 35)	F1 (*n* = 12)	F2 (*n* = 5)	F3 (*n* = 5)	F4 (*n* = 9)	*p*
Vitamin A, µmol/L, median	1.69	1.63	1.98	1.77	1.89	0.41
Vitamin E, µmol/L, median	11.17	30.91	14.19	7.96	3.39	0.29
E:C ratio, median	2.30	5.23	3.61	2.46	0.76	0.21
E:TG ratio, median	8.97	20.28	14.96	18.56	5.04	0.31
E:C < 3.7, *n* (%)	23/34 (67.6)	4/12 (33.3)	3/5 (60.0)	3/5 (60.0)	7/8 (87.5)	0.14

*p*-values from Kruskal–Wallis test for continuous variables (*n* = 66 with transient elastography for all) and Fisher exact test for the categorical row (*n* = 64; two patients had transient elastography but no E:C ratio available). Cells for E:C < 3.7 report the number of children with a low ratio/number of children with available data (%).

## Data Availability

The de-identified dataset used for this analysis is available from the corresponding author on reasonable request.

## Abbreviations

25(OH)D	25-hydroxyvitamin D
CALIPER	Canadian Laboratory Initiative on Pediatric Reference Intervals
CI	Confidence interval
CLD	Chronic liver disease
E:C	Vitamin E-to-total cholesterol ratio
ELISA	Enzyme-linked immunosorbent assay
ESPGHAN	European Society for Pediatric Gastroenterology, Hepatology and Nutrition
E:TG	Vitamin E-to-triglyceride ratio
GGT	Gamma-glutamyltransferase
HPLC	High-performance liquid chromatography
IQR	Interquartile range
METAVIR	Histological liver fibrosis scoring system (METAVIR)
NASPGHAN	North American Society for Pediatric Gastroenterology, Hepatology and Nutrition
OR	Odds ratio
RBP4	Retinol-binding protein 4
TMB	Tetramethylbenzidine
WHO	World Health Organization
